# Supplementary material for: HyperG-PS: Voxel correlation modeling via hypergraph for LiDAR panoptic segmentation
Source: Fundam Res. 2025 Jan 26;6(3):1727–35. doi: 10.1016/j.fmre.2024.03.033 (PMC13247466; doi:10.1016/j.fmre.2024.03.033)
Supplement: Supplementary Data S1 — Supplementary Raw Research Data. This is open data under the CC BY license http://creativecommons.org/licenses/by/4.0/ [file mmc1.docx]

%%

%% Copyright 2007-2020 Elsevier Ltd

%%

%% This file is part of the 'Elsarticle Bundle'.

%% ---------------------------------------------

%%

%% It may be distributed under the conditions of the LaTeX Project Public

%% License, either version 1.2 of this license or (at your option2 any

%% later version. The latest version of this license is in

%% http://www.latex-project.org/lppl.txt

%% and version 1.2 or later is part of all distributions of LaTeX

%% version 1999/12/01 or later.

%%

%% The list of all files belonging to the 'Elsarticle Bundle' is

%% given in the file `manifest.txt'.

%%

%% Template article for Elsevier's document class `elsarticle'

%% with numbered style bibliographic references

%% SP 2008/03/01

%%

%%

%%

%% $Id: elsarticle-template-num.tex 190 2020-11-23 11:12:32Z rishi $

%%

%%

\documentclass[preprint,12pt]{elsarticle}

%% Use the option review to obtain double line spacing

%% \documentclass[authoryear,preprint,review,12pt]{elsarticle}

%% Use the options 1p,twocolumn; 3p; 3p,twocolumn; 5p; or 5p,twocolumn

%% for a journal layout:

%% \documentclass[final,1p,times]{elsarticle}

%% \documentclass[final,1p,times,twocolumn]{elsarticle}

%% \documentclass[final,3p,times]{elsarticle}

%% \documentclass[final,3p,times,twocolumn]{elsarticle}

%% \documentclass[final,5p,times]{elsarticle}

%% \documentclass[final,5p,times,twocolumn]{elsarticle}

%% For including figures, graphicx.sty has been loaded in

%% elsarticle.cls. If you prefer to use the old commands

%% please give \usepackage{epsfig}

%% The amssymb package provides various useful mathematical symbols

\usepackage{amssymb}

\usepackage{amsmath}

\usepackage{amsfonts}

\usepackage{color}

\usepackage{booktabs}

%% The amsthm package provides extended theorem environments

%% \usepackage{amsthm}

\definecolor{sqColor}{rgb}{1,0.0,0.0}

\newcommand{\sq}[1]{{\color{sqColor} #1}}

%% The lineno packages adds line numbers. Start line numbering with

%% \begin{linenumbers}, end it with \end{linenumbers}. Or switch it on

%% for the whole article with \linenumbers.

%% \usepackage{lineno}

\journal{Fundamental Research}

% \usepackage{algpseudocode}

% \usepackage{algorithm}

\usepackage[linesnumbered,ruled]{algorithm2e}

\begin{document}

\newsavebox\CBox

\def\textBF#1{\sbox\CBox{#1}\resizebox{\wd\CBox}{\ht\CBox}{\textbf{#1}}}

\begin{frontmatter}

%% Title, authors and addresses

%% use the tnoteref command within \title for footnotes;

%% use the tnotetext command for theassociated footnote;

%% use the fnref command within \author or \address for footnotes;

%% use the fntext command for theassociated footnote;

%% use the corref command within \author for corresponding author footnotes;

%% use the cortext command for theassociated footnote;

%% use the ead command for the email address,

%% and the form \ead[url] for the home page:

%% \title{Title\tnoteref{label1}}

%% \tnotetext[label1]{}

%% \author{Name\corref{cor1}\fnref{label2}}

%% \ead{email address}

%% \ead[url]{home page}

%% \fntext[label2]{}

%% \cortext[cor1]{}

%% \affiliation{organization={},

%% addressline={},

%% city={},

%% postcode={},

%% state={},

%% country={}}

%% \fntext[label3]{}

\title{\textcolor{black}{HyperG-PS: Voxel Correlation Modeling via Hypergraph for LiDAR Panoptic Segmentation}}

%% use optional labels to link authors explicitly to addresses:

%% \author[label1,label2]{}

%% \affiliation[label1]{organization={},

%% addressline={},

%% city={},

%% postcode={},

%% state={},

%% country={}}

%%

%% \affiliation[label2]{organization={},

%% addressline={},

%% city={},

%% postcode={},

%% state={},

%% country={}}

% \author{Lin Bie\fnref{label2}}

% \author{Yipeng Li\fnref{label2}}

% \author{Gang Xiao\fnref{label2}}

% \author{Yue Gap\corref{cor1}\fnref{label2}}

\author[1]{Lin Bie}

\author[2]{Gang Xiao}

\author[3]{Yipeng Li}

\author[1]{Yue Gao}

%\author[1,2]{\fnm{Lin} \sur{Bie}}

%\author[1,2]{\fnm{Gang} \sur{Xiao}}

%\author[1,3]{\fnm{Yipeng} \sur{Li}}

%\author[1,3]{\fnm{Yue} \sur{Gao}}

% \affil[1]{\orgdiv{\small{BNRist, THUIBCS, BLBCI}}, \orgname{Tsinghua University}, \orgaddress{\city{Beijing} \postcode{100084}, \country{China}}}

% \affil[2]{\orgdiv{\small{KLISS, School of Software}}, \orgname{Tsinghua University}, \orgaddress{\city{Beijing} \postcode{100084}, \country{China}}}

% \affil[3]{\orgdiv{\small{Department of Automation}}, \orgname{Tsinghua University}, \orgaddress{\city{Beijing} \postcode{100084}, \country{China}}}

\affiliation[1]{organization={BNRist, THUIBCS, KLISS, BLBCI, School of Software,Tsinghua University},%Department and Organization

city={Beijing},

postcode={100084},

country={China}}

\affiliation[2]{organization={Jiangxi KMAX Industrial Co.,

Ltd.},%Department and Organization

city={Nanchang},

postcode={330100},

country={China}}

\affiliation[3]{organization={THUIBCS, Department of Automation, Tsinghua University},%Department and Organization

city={Beijing},

postcode={100084},

country={China}}

\begin{abstract}

%% Text of abstract

Light-detection-and-ranging (LiDAR) point-cloud panoptic segmentation is a fundamental task in autonomous driving because it integrates the tasks of static environmental understanding and dynamic object identification, which have recently gained significant research interest.

% In this paper, we proposed a hypergraph-based bottom-up panoptic segmentation framework named HyperG-PNet. As a novelty network, HyperG-PNet concentrates on improving cluster performance in panoptic segmentation which is the core problem in LiDAR instance segmentation task. To tackle this, we design a cross-modality features extraction network which fuses the 3D point cloud features and 2D BEV features at voxel level. We utilize hypergraph to model the relationship between instance and voxels which bridges the gap between voxels features and instance label.

In this paper, we propose a bottom-up panoptic-segmentation framework based on hypergraph learning, named HyperG-PS, which addresses the core problem of LiDAR panoptic segmentation by improving the cluster performance of instance segmentation,. Our framework inputs the raw LiDAR point cloud and uses a multi-view feature-extraction network to fuse the 3D point-cloud features and 2D BEV features at the voxel level. Afterward, we model the correlation among voxels using a hypergraph to bridge the gap between voxel features and instance labels. We enhance the representation of a voxel, thus improving the cluster algorithm performance while directly avoiding predicting point cloud offsets by using our hypergraph-learning module. Extensive experiments on the SemanticKITTI and nuScenes datasets demonstrated the superior performance of HyperG-PS compared with state-of-the-art methods.

\end{abstract}

%%Graphical abstract

%\begin{graphicalabstract}

%\includegraphics{grabs}

%\end{graphicalabstract}

%%Research highlights

%%\begin{highlights}

%%\item Research highlights 1

%%\item Research highlights 2

%%\end{highlights}

\begin{keyword}

%% keywords here, in the form: keyword \sep keyword

%% PACS codes here, in the form: \PACS code \sep code

%% MSC codes here, in the form: \MSC code \sep code

%% or \MSC[2008] code \sep code (2000 is the default)

Semantic Segmentation, Panoptic Segmentation, LiDAR Point-Cloud Voxelization, Hypergraph Learning.

\end{keyword}

\end{frontmatter}

%% \linenumbers

%% main text

\section{Introduction}

The recent rapid development of autonomous driving requires unmanned vehicles to operate in challenging open environments with a variety of objects, making comprehensive perception a challenging task for intelligent transportation. Benefiting from the recently proposed instance-level labeled scan datasets, panoptic segmentation promotes environment-perception research for autonomous vehicles. In particular, the panoptic-segmentation task aims to recognize object instances in a complicated urban environment by unifying semantic and instance segmentation in a single architecture~\cite{panoptic}; \textit{} that is, it requires the model to be able to identify the semantics of both the background (``stuff" class) and instance of the foreground (``thing" class).

Panoptic-segmentation approaches can be proposal-based and proposal-free, depending on whether or not they use an independent network to obtain object proposals. In the early stages, most methods predict proposals through object detection~\cite{kitti, yang, gspn}. The primary weakness of these proposal-based methods consists in the fact that their accuracy relies heavily on the performance of object detection. On the contrary, some proposal-free methods~\cite{DSN, pups, lps} have been proposed, with a focus on improving the performance of cluster operations during instance segmentation. Since these proposal-free methods are not affected by other independent subtasks, \textit{e.g.}, object detection, they have become increasingly popular in recent years owing to their relatively concise structure. As the core part of proposal-free methods, the clustering algorithm in the high-dimension feature space is significant for the instance-segmentation task. Nevertheless, widely used heuristic clustering algorithms (\textit{e.g.}, Mean Shift~\cite{MS} and HDBSCAN~\cite{HDBS}) have disadvantages in local optimization and time consumption. Apparently, clustering effectiveness and efficiency are critical to proposal-free methods for panoptic-segmentation performance.

Compared with the well-studied 2D image and 3D indoor point-cloud segmentation tasks, 3D panoptic segmentation is in its infancy and remains a challenge, mainly owing to the sparsity and irregularity of the underlying light-detection-and-ranging (LiDAR) data. \textcolor{black}{Since the point-cloud segmentation task is greatly dependent on spatial features, encoder–decoder architectures are widely employed to construct relationships between specific points and their semantic labels from point clouds~\cite{polarnet}.} In contrast to object-classification tasks, the spatial-position feature of the point cloud plays a more critical role than shape features in panoptic-segmentation tasks. Hence, applying sparse convolution on the LiDAR point cloud directly is not the best choice for outdoor 3D panoptic-segmentation tasks for data distribution. In other words, the shape feature of the incomplete point cloud may not be as important as the position feature, but it still determines instance boundaries. \textcolor{black}{As a result, owing to the sparsity and irregularity of the LiDAR point cloud, voxel-based representation learning can better extract features from few points from distant instances~\cite{polarnet,MultiNet}. These methods have advantages with regard to position utilization and information distribution. Moreover, voxelized point clouds are suitable for further tackling panoptic tasks by using over-segmented methods in subsequent stages.}

% these methods have the advantage of decreasing computational complexity and time consumption, which is crucial in autonomous driving. Thus, the panoptic segmentation problem is transformed into a conventional voxel-wise feature clustering problem.

In the panoptic-segmentation task, the relationship between the point cloud and instance can be naturally modeled by a graph structure. Recently a novel graph-based clustering method~\cite{GP} was proposed to predict instances from over-segmented clusters effectively. However, compared with the graph, the hypergraph has excellent capability to model the correlation between them~\cite{feng_hgnn}. Its performance is also constrained by applying sparse convolution on the LiDAR point cloud directly. Another work~\cite{PHNet} based on voxelization and multi-view feature fusion showed good performance by introducing the KNN-transformer to predict offset regression. However, it neglects to model spatial relationships among different voxels, which results in relatively weak performance at the instance-segmentation period. Some recent methods~\cite{lps,rangenet++} project point clouds into the 2D plate to conduct 2D feature representation by using a CNN-based network. The 2D projection provides a multi-view feature to fuse, which has better global contextual information. \textcolor{black}{However, the authors selected the range-map view (RV) as the plate to project, which resulted in location relationship information missing among point clouds. In summary, the LiDAR panoptic-segmentation task faces two main challenges:}\begin{itemize}

\item \textcolor{black}{Owing to the sparsity and irregularity of LiDAR point clouds, prior works have been unable to adequately learn both spatial and shape features simultaneously;}

\item \textcolor{black}{Existing methods fail to effectively model the correlation between voxel features and their corresponding instance labels.}

\end{itemize}

% Compared to well-studied 2D image and 3D indoor point clouds segmentation, 3D panoptic segmentation is by and large at its infancy and still very challenging due to sparsity and irregularity of input LiDAR data. Because segmentation task largely relies on spatial feature, methods of sparse convolution and encoder-decoder structure are widely used to conduct feature extraction of point cloud. Unlike other classification tasks, the spatial position feature of cloud point plays more important roles than shape feature in panoptic segmentation. Therefore voxel-based representation learning of point cloud methods are popular for their advantage in saving memory consumption and making fully use of spatial position information. On the other hand, point cloud can be projected into 2D space and employ the 2D feature extraction architecture such as Convolutional Neural Networks (CNNs).

%%\sq{Compared to traditional 2D image or 3D indoor point cloud semantic segmentation task, 3D panoptic segmentation is a relatively new and challenging task due to the sparsity and irregularity of the input LiDAR data. Since the segmentation task mainly relies on spatial feature, methods of sparse convolution and encoder-decoder structure are widely used to conduct feature extraction of point cloud.

%%Different from object classification task, the spatial position feature of the point cloud plays more important roles than shape feature in panoptic-segmentation task. Therefore, voxel-based representation learning methods are more widely used due to their advantages in terms of less memory consumption and full utilization of spatial location information.

%%Obviously, clustering effectiveness and efficiency are critical to the final result. Although Panoptic-PolarNet attempts to use KNN-Transformer algorithms to enhance accuracy of clustering, transformer structure may not be suitable to model spatial correlation between points.

%To tackle these challenges, we propose a pipeline to fuse 2D features from BEV branch and 3D features from voxel-based branch that contains both voxel-level and pixel-level information. Specifically, this architecture can fully exploit local geometrical relations and avoid huge computing consumption in 3D point cloud convolution at the same time. Instead of obtaining cluster offset through cluster operation directly, we introduce hypergraph conventional neural network(HGNN) to model voxel spatial correlation. Unlike GP-S3net, we utilize hyperedge to indicate voxels belong to single instance which can better describe their hidden common feature than subgraph. Through HGNN representation learning process, voxels feature also turn better to improve final cluster result. We also use equivalent operation to accelerate traditional cluster during hypergraph construction. We evaluate our Hypergraph-based panoptic-segmentation network on SemanticKITTI~\cite{kitti}. The experimental results show that our proposed method achieves state-of-the-art performance.

\textcolor{black}{To address these challenges, we propose an efficient voxel-correlation modeling framework that is based on hypergraph learning.} The proposed method fuses 2D features extracted from the BEV branch and 3D features extracted from the voxel-based branch to fully utilize both voxel-level and pixel-level information to achieve efficient panoptic segmentation. In contrast to methods projecting point clouds to an RV map, the BEV projection approach adopted in this study zeroes out the height dimension of the LiDAR point cloud, rather than the depth dimension. Not only does this approach avoid overlap between different object instances, but it also allows us to exploit local geometric relationships fully. \textcolor{black}{Moreover, the method proposed in this paper reduces the computational cost compared with conducting 3D convolution operations on each point cloud direct method.} Rather than obtaining cluster offset through cluster operation directly, we leverage a hypergraph conventional neural network (HGNN) ~\cite{gao_hgnn} to model the high-order voxel-spatial correlation. In contrast to existing graph-based methods, we utilize hyperedge to indicate voxels that belong to a single instance. This approach enables us to better model the hidden relational features between voxels than methods using subgraphs~\cite{GP}. Using HGNN representation learning, voxel features can be enhanced to improve the final clustering performance. An equivalent operation is leveraged to accelerate the traditional clustering during hypergraph construction. We evaluated our proposed method on SemanticKITTI~\cite{kitti} and nuScenes~\cite{nuscenes}datasets. The experimental results demonstrate that our proposed method achieved better performance than state-of-the-art methods.

Our contribution can be summarized as follows:

\begin{itemize}

\item \textcolor{black}{We introduce a novel initiative for LiDAR panoptic segmentation that models the correlations among voxels and instance labels using hypergraph learning, which can significantly improve instance-segmentation performance. Ablation experiments showed the hypergraph-based method's superiority to the simple graph-based method;}

\item \textcolor{black}{We propose a framework that can seamlessly fuse 3D LiDAR data features with 2D BEV features from separate benches by voxelizing the point cloud. This approach improves LiDAR panoptic-segmentation performance compared with prior networks utilizing only a single-view branch;}

\item \textcolor{black}{Extensive experiments indicate the efficacy of our proposed framework, which achieved strong performance on the SemanticKITTI~\cite{kitti} and nuScenes~\cite{nuscenes} datasets.}

\end{itemize}

\section{Related Work}

\subsection{LiDAR Semantic Segmentation}

LiDAR point-cloud semantic segmentation plays a fundamental role in panoptic-segmentation tasks and has attracted significant attention owing to its wide use in many fields (\textit{e.g.,} autonomous driving). Depending on the different ways to preprocess this sparse and irregular LiDAR point cloud; semantic-segmentation approaches can be divided into three categories—projection-based, point-based, and voxel-based methods. Projection-based methods transform the 3D LiDAR sparse point cloud into either 2D BEV ~\cite{polarnet,rt3d}, or an RV map ~\cite{lps,rangenet++,lite}. The traditional way to project a point cloud into an RV map zeroes out the depth, while recent methods prefer using BEV by zeroing out the point-cloud height. Since the LiDAR view is 360 degrees, these BEV-based methods can obtain better global 2D features to fuse with and overcome the weakness of information loss during projection. Point-based methods are developed together with the 3D sparse CNN~\cite{mink,3dsparse}, which provides the possibility to obtain features from the point cloud directly. Nevertheless, it is difficult to use these methods in outdoor situations. Voxel-based methods transform sparse point clouds into voxels, which can fundamentally decrease 3D-convolution computational requirements.

\subsection{LiDAR Panoptic Segmentation}

Panoptic segmentation unifies both semantic segmentation and instance segmentation. Compared with indoor point-cloud panoptic segmentation, LiDAR point-cloud panoptic segmentation is a newly proposed task owing to the difficulty in labeling data. SemanticKITTI~\cite{semantickitti} is the first panoptic segmentation and most widely used LiDAR point-cloud dataset, followed by nuScene~\cite{nuscenes}, Waymo~\cite{waymo}, and SemanticPOSS~\cite{poss}. Based on the sequence of data-processing frameworks, LiDAR panoptic segmentation can be proposal-based and proposal-free methods. Previous studies~\cite{s3net, yang2019learning} have mainly focused on proposal-based panoptic segmentation to attach the semantic head to classify ``stuff" points, such as SGPN~\cite{sgpn} and panoptic FPN~\cite{fpn}. These methods usually are two-stage and work by assembling an object detector and a semantic-segmentation network, which is also known as the top-bottom work stream. Apparently, the performance of proposal-based approaches is heavily dependent on the object-detection result. In contrast, proposal-free methods~\cite{pups, PHNet} attempt to predict instances directly, without conducting object detection tasks. These bottom-top approaches are first utilized by DeeperLab~\cite{cheng_panoptic} in 2D panoptic segmentation and are introduced to LiDAR panoptic segmentation by Panotic-PolarNet~\cite{polarnet}. Compared with proposal-based panoptic-segmentation methods, which are prone to class and instance inconsistency, proposal-free approaches predict the instance label directly, thereby making the instance ID's information more accessible to attach to the point-cloud feature.

\subsection{Representation Learning of a LiDAR Point Cloud}

Since the result of the LiDAR panoptic-segmentation task is primarily affected by the efficiency of sparse point-cloud representation, previous studies~\cite{pointnet, kpconv} have focused on two ways to enhance learning-based data representation.\textcolor{black}{ One is to improve the performance of feature learning on the point cloud directly, by using a more powerful network---such as RS-CNN~\cite{liu2019rscnn}, PointConv~\cite{wu2019pointconv}, and point transformer~\cite{zhao2021pointtrans}---as a 3D branch backbone.} However, this type of method has the advantage of learning local features for small regular objects and the disadvantage of extracting global features for large-scale outdoor environments. Voxel-based approaches, such as VoxelNet~\cite{voxelnet} and SECOND~\cite{second}, perform better on sparse and irregular data by introducing sparse convolution to promote learning efficiency. In contrast, recent studies~\cite{GP,liang20203d} have started to leverage the graph to model the spatial correlation between the different clusters of point clouds to enhance global feature representation. Compared with the graph structure, the hypergraph~\cite{feng_hgnn} has good ability to model the internal relationships of the point cloud owing to its far more flexible structure. Recent work has shown that hypergraph~\cite{gao_hgnn} achieves superior performance in point-cloud representation by bridging semantic features and spatial features.

\section{Method}

%==================fig==================%

\begin{figure*}[t]

\center

\includegraphics[width=1\textwidth]{picture/pic1-pipeline.png}

\vspace{-0.3cm}

\caption{Our proposed method, HyperG-PS, takes both 2D and 3D features from BEV projection and voxelization of the point cloud as input. The approach uses MLP to extract 2D and 3D features, which are then fused together with a fusion layer. The resulting fused features are fed into a Unet-based backbone to predict semantic segmentation, which provides a ``thing" mask for the panoptic task. A hypergraph-learning module is leveraged to enhance the representation of the voxel, and this benefits the final cluster performance. The final output is a combination of the semantic segmentation and instance ID's prediction as the panoptic segmentation.}

\vspace{-0.3cm}

\label{fig1-pipeline}

\end{figure*}

%=======================================%

% In this section, we first present a brief of our proposed Hyper-based panoptic-segmentation network and detail each of its constituting components. The framework of our HPSNet is shown in Figure~\ref{fig1-pipeline}.

This section first presents a brief overview of our proposed hypergraph-based panoptic-segmentation network, known as HyperG-PS, whereupon the detailed components of our proposed method are introduced. The framework of our HyperG-PS is shown in Figure~\ref{fig1-pipeline}.

\subsection{Overview}

In order to establish a voxel index to balance computation consumption and efficacy in feature representation, we first voxelize the LiDAR point-cloud coordinates. At the same time, we project the point cloud onto BEV~\cite{polarnet} to obtain 2D features at the pixel level by constructing a max-pooling multilayer perception (MLP) backbone.

The BEV image appears to lose the z-axis information in the urban scene and still retains the original LiDAR irregular and sparse pattern, making it unsuitable for CNN encoders. To address this, we utilize polar coordinates based on the cylinder3D~\cite{cylinder3d} to replace Cartesian coordinates, making 2D features more suitable for learning with an MLP structure. To facilitate the fusion of features with the 2D branch, we employ a voxel-based MLP encoder~\cite{cylindrical} in the 3D branch . After both branches finish their representation learning, we employ a feature-fusion layer to narrow the multiview-feature gap and make the training process more manageable. The new features comprise both shape and position information at the voxel level, which we feed into the semantic-segmentation branch and instance-segmentation branch, respectively. The semantic branch provides a ``thing" mask for instance segmentation, thus significantly reducing clustering and hypergraph learning computation. In the instance segmentation branch, we conduct K-nearest neighbors on voxels to construct a hypergraph structure. Voxels classified into one cluster are then connected by a hyperedge. We use hypergraph to model the interaction among ``thing" voxels and conduct hypergraph learning to enhance their feature representation using instance IDs' labels as supervised information. Another cluster operation is used to predict the instance IDs for each voxel. Finally, we combine the outputs of instance segmentation and semantic segmentation to obtain the final panoptic-segmentation results.

\subsection{Hypergraph Construction}

The details of the hypergraph construction are introduced in this section. The hypergraph structure has been introduced into many computer-vision fields owing to its superior performance in complex relation modeling. In the panoptic-segmentation task, the correlation between ``thing," instance, and voxel cluster naturally has an inclusion relationship that is perfect for the mode by graph or hypergraph structure. Compared with the graph structure, hypergraph was chosen not only for its strong ability in feature enhancement but also for avoiding conducting many iterative operations that change the graph structure directly. Our framework uses a voxel with fused features as the basic unit and vertex on the hypergraph. A hypergraph comprises several weighted hyperedges, each containing multiple vertices. Hyperedge is better at modeling multiple voxels in one instance relationship than the simple graph where an edge only connects two vertices.

We begin by applying the KNN algorithm to the voxel features to construct the hypergraph. For each voxel, we find its K-nearest neighbors and form a hyperedge that connects these voxels. The weight of the hyperedge is calculated using a learned hyperedge weight function that takes the features of all voxels in the hyperedge as input. This weight function can capture the high-order interactions among voxels in the hyperedge.

%==================fig==================%

\begin{figure*}[t]

\center

\includegraphics[width=1\textwidth]{picture/pic2-hlm.png}

\vspace{-0.3cm}

\caption{Illustration of the hypergraph-learning module. The input of the module is fine-grained voxel features belonging to ``thing" classes according to semantic segmentation. In the module, the KNN method is employed to construct a hypergraph to further improve the representation of the voxels. Instance ID labels are used as hyperedge supervision information for the hypergraph learning process. The output of the module is the cluster result of the enhanced voxel features.}

\vspace{-0.3cm}

\label{fig2-hlm}

\end{figure*}

%=======================================%

Based on the fused features of each voxel $\mathbf{F} = \{ {f_1},{f_2},...,{f_K}\}$, we can construct the hypergraph $\mathcal{G} = (\mathcal{V},\mathcal{E},\mathbf{W})$, where $\mathcal{V}$ is the set vertices in the graph (i.e., the features $\mathbf{F}$), $\mathcal{E}$ represent the set of edges, and the diagonal matrix $\mathbf{W}$ corresponds to the edge weights. In our methods, we leverage widely used KNN-based method cluster algorithms to construct hyperedges. To be precise, each time, we select one vertex $v$ in $\mathcal{V}$ as the centroid, find its $K$-nearest neighbors $\mathcal{K}(v)$ by calculating Euclidean distances, and then put them into a hyperedge. For a convenient representation of the hypergraph $\mathcal{G}$, we define a $\left| \mathcal{V} \right| \times \left| \mathcal{E} \right|$ incidence matrix $\mathbf{H}$:

\begin{equation}

\mathbf{H}(v,e) = \left\{\begin{array}{l}

\begin{array}{*{20}{c}}1&{{\rm{if}}\ v \in e}

\end{array}\\

\begin{array}{*{20}{c}}0&{{\rm{if} }\ v \notin e}

\end{array}

\end{array}\right.

\end{equation}

For a vertex for the $v\in \mathcal{V}$, its degree is defined by $d(v) = \sum\nolimits_{e \in E} {\mathbf{W}(e)\mathbf{H}(v,e)}$. For a hyperedge $e\in \mathbf{E}$, its degree is defined by $\delta (e) = \sum\nolimits_{v \in V} {\mathbf{H}(v,e)} $. Further, ${\mathbf{D}_e}$ and ${\mathbf{D}_v}$ denote the diagonal matrices of the hyperedge degrees and vertex degrees, respectively. To simplify the calculation, we initialize $\mathbf{W}$ as an identity matrix, signifying equal weights for all hyperedges. We then input a cluster composed of voxels to the global-average pooling layer and obtain the embedding vector as hyperedge features that have the same dimension as the vertex.

\subsection{Hypergraph Learning}

In this section, we introduce the details of how hypergraph learning enhances the representation of voxels, as shown in Figure~\ref{fig2-hlm}. After constructing the hypergraph, we perform hypergraph learning to enhance the representation of voxel features. In particular, we use the instance IDs obtained from the clustering algorithm supervised by the ground-truth labels to train the hypergraph. The hypergraph-learning module aims to learn a matrix that enhances the original voxel representation to make the voxels with the same ID label closer. This matrix is learned by minimizing a hypergraph-based loss function that considers the similarity among voxels in the same hyperedge and the dissimilarity among voxels in different hyperedges. Through the hypergraph-learning module, the representation of voxel features is enhanced, and the correlation among voxels in the same instance is better modeled. This improves the performance of the clustering algorithm, leading to more accurate instance-segmentation results.

Considering the constructed hypergraph $\mathcal{G} = (\mathcal{V},\mathcal{E},\mathbf{W})$, we first generate a correlation matrix $\mathbf{L}$:

\begin{equation}

\mathbf{L} = {\mathbf{D}_v}^{ - {1 \mathord{\left/

{\vphantom {1 2}} \right.

\kern-\nulldelimiterspace} 2}}\mathbf{H}\mathbf{W}{\mathbf{D}_e}^{ - 1}{\mathbf{H^T}}{\mathbf{D}_v}^{ - {1 \mathord{\left/

{\vphantom {1 2}} \right.

\kern-\nulldelimiterspace} 2}},

\end{equation}

$L$ describes the vertex correlations in the hypergraph.

Subsequently, we input the correlation matrix $\mathbf{L} \in {R^{{N^ * } \times {N^ * }}}$ and the features $\mathbf{F} = \{ {f_1},{f_2},...,{f_K}\} \in {R^{{N^ * } \times {K }}}$ into the hypergraph-based panoptic-segmentation network. ${N^ * }$ denotes the number of voxels in the ``thing" class. Moreover, the layer-wise propagation rule of our quality predictor is as follows:

\begin{equation}

{\mathbf{X}^{(t + 1)}} = \sigma (B{N_{\gamma ,\beta }}(\mathbf{L}{\mathbf{X}^{(t)}}\Theta _h^{(t)})),

\end{equation}

where $\sigma$ is the ReLU activation function. $\mathbf{X}^{(t+1)}$ is the output of the $t$-th layer. ${\mathbf{X}^{(0)}} = \mathbf{F}$ and are the outputs of the last hypergraph convolution layer is ${ \mathbf{X}^{(n)}} \in {R^{{N^ * } \times {N^ * }}}$ with new features. $\Theta _h^{(t)}$ denotes the learnable parameters in the $t$-th layer. $B{N_{\gamma ,\beta }}$ is batch normalization, where $\gamma$ and $\beta$ are learnable parameters. In practice, we utilize a two-layer hypergraph-learning structure to speed up the training. \textcolor{black}{The detailed pseudo-code of this process can be found in Algorithm 1. }

%==================pseudo-code==================%

\renewcommand{\thealgocf}{1}

\begin{algorithm}

% \State $c:=KNN(f);$

% \State $l_hyperedge:=zeros(N,N)$

\SetAlgoLined %显示end

\caption{\textcolor{black}{An algorithm on hypergraph learning}}

\SetKwInOut{Input}{Input}

\SetKwInOut{Output}{Output}

% Input

\textcolor{black}{\Input{Voxel features $f \in {\mathbf{F}^{N \times F'}}$, things instance label $l \in {L^N}$;}}

% Output

\textcolor{black}{\Output{Enhanced voxel features $f' \in {{\hat F}^{N \times F'}}$;}}

% $$括起来表示这是一个数学表达式,会使用英文斜体表示. \\表示换行

\textcolor{black}{$C:=KNN(f);$ }\\

\textcolor{black}{${l_{hyperedge}}:=zeros(C,N)$} \\

\textcolor{black}{\If{${f_j} \in {C_i}$} {

$l_hyperedge(i,j):=1$

}}

\textcolor{black}{Hypergraph $\mathcal{G}$ incidence matrix: $\mathbf{H}(v,e) ={{l_{hyperedge}}}(i,j),i \in C,j \in N $} \\

\textcolor{black}{\For{$j = 1,2, \ldots ,N$ }{

$d(v_j) \leftarrow \sum\nolimits_{e \in E} {\mathbf{W}(e)\mathbf{H}(v_j,e)} $, $\delta (e_j) = \sum\nolimits_{v \in V} {\mathbf{H}(v,e_j)} $ \\

}

${\mathbf{D}_v} \leftarrow diag(d({v_1}), \ldots ,d({v_N})), {\mathbf{D}_e} \leftarrow diag(d({e_1}), \ldots ,d({e_N})); $ \\

Hypergraph Laplacian matrix:$ \mathbf{L} \leftarrow {\mathbf{D}_v}^{ - {1 \mathord{\left/

{\vphantom {1 2}} \right.

\kern-\nulldelimiterspace} 2}}\mathbf{H}\mathbf{W}{\mathbf{D}_e}^{ - 1}{\mathbf{H^T}}{\mathbf{D}_v}^{ - {1 \mathord{\left/

{\vphantom {1 2}} \right.

\kern-\nulldelimiterspace} 2}}$; \\

Initialize: $\mathbf{X^{(0)}} \leftarrow \mathbf{F}^{N \times F'};$ \\

\For{$t = 0,1$ }{

${\mathbf{X}^{(t + 1)}} \leftarrow \sigma (B{N_{\gamma ,\beta }}(\mathbf{L}{\mathbf{X}^{(t)}}\Theta _h^{(t)})) $ \\

}

\KwRet $f' \in {{\hat F}^{N \times F'}}$}

\end{algorithm}

%==================pseudo-code==================%

% %==================pseudo-code==================%

% \renewcommand{\thealgocf}{1}

% \begin{algorithm}

% % \State $c:=KNN(f);$

% % \State $l_hyperedge:=zeros(N,N)$

% \SetAlgoLined %显示end

% \caption{\textcolor{blue}{An algorithm on hypergraph learning}}

% \SetKwInOut{Input}{Input}

% \SetKwInOut{Output}{Output}

% % Input

% \textcolor{blue}{\Input{Voxel features $f \in {\mathbf{F}^{N \times F'}}$, things instance label $l \in {L^N}$;}}

% % Output

% \textcolor{blue}{\Output{Enhanced voxel features $f' \in {{\hat F}^{N \times F'}}$;}}

% % $$括起来表示这是一个数学表达式,会使用英文斜体表示. \\表示换行

% \textcolor{blue}{$C:=KNN(f);$ }\\

% \textcolor{blue}{${l_{hyperedge}}:=zeros(C,N)$} \\

% \textcolor{blue}{\If{${f_j} \in {C_i}$} {

% $l_hyperedge(i,j):=1$

% }}

% \textcolor{blue}{Hypergraph $\mathcal{G}$ incidence matrix: $\mathbf{H}(v,e) ={{l_{hyperedge}}}(i,j),i \in C,j \in N $} \\

% \textcolor{blue}{\For{$j = 1,2, \ldots ,N$ }{

% $d(v_j) \leftarrow \sum\nolimits_{e \in E} {\mathbf{W}(e)\mathbf{H}(v_j,e)} $, $\delta (e_j) = \sum\nolimits_{v \in V} {\mathbf{H}(v,e_j)} $ \\

% }

% ${\mathbf{D}_v} \leftarrow diag(d({v_1}), \ldots ,d({v_N})), {\mathbf{D}_e} \leftarrow diag(d({e_1}), \ldots ,d({e_N})); $ \\

% Hypergraph Laplacian matrix:$ \mathbf{L} \leftarrow {\mathbf{D}_v}^{ - {1 \mathord{\left/

% {\vphantom {1 2}} \right.

% \kern-\nulldelimiterspace} 2}}\mathbf{H}\mathbf{W}{\mathbf{D}_e}^{ - 1}{\mathbf{H^T}}{\mathbf{D}_v}^{ - {1 \mathord{\left/

% {\vphantom {1 2}} \right.

% \kern-\nulldelimiterspace} 2}}$; \\

% Initialize: $\mathbf{X^{(0)}} \leftarrow \mathbf{F}^{N \times F'};$ \\

% \For{$t = 0,1$ }{

% ${\mathbf{X}^{(t + 1)}} \leftarrow \sigma (B{N_{\gamma ,\beta }}(\mathbf{L}{\mathbf{X}^{(t)}}\Theta _h^{(t)})) $ \\

% }

% \KwRet $f' \in {{\hat F}^{N \times F'}}$}

% \end{algorithm}

% %==================pseudo-code==================%

\subsection{Backbone Architecture}

In our 2D feature extraction branch, we transform the origin coordinates into BEV coordinates based on PolarNet~\cite{polarnet}. After projecting a 3D point cloud to a 2D BEV, we adopt an MLP encoder to obtain pixel-level features of the BEV image. In contrast to normal images where each pixel consists of RGB, the grid of the BEV image obtained by projection is binary, which is more suitable for MLP than CNN-based methods. Next, a max-pooling layer is used at each BEV grid to create a representation of $M \in {R^{H \times W \times K_{2D}}}$, where $K_{2D}$ is the number of feature channels. We take $K_{2D} = 64$ in our experiment.

In the voxelization branch, we first categorize the LiDAR point cloud into voxels with cylindrical-space partition~\cite{cylinder3d}. According to the position and shape, point-cloud features are represented as $(N \times {N_P}^ * ) \times {K_{3D}}$, where $N$ is the number of non-empty voxels, and ${N_P}^ *$ is point number in each voxel, and $K_{3D}$ is the feature dimensions. We utilize a three-layer MLP with batch normalization and ReLU to obtain feature representation. As in the 2D branch, we apply a max-pooling layer to maintain the consistency of features for each voxel. To accelerate the training, we reduce the feature dimension of the voxel to 64 with a single-layer MLP and obtain the $V \in {R^{N \times 64}}$.

We fuse the features from both the 2D and 3D branches as the final point-cloud representation. Specifically, we map the voxel features $V \in {R^{N \times 64}}$ to the polar BEV coordinate $M \in {R^{H \times W \times 64}}$ and perform fusion feature with the shape ${F_i} \in {R^{(H \times W \times {N_v}^ * ) \times K}}$, where ${N_v}^ *$ denotes the number of voxels in each BEV grid. Then, we adopt MLP and max-pooling layer to get the $H \times W \times 64$ for fused feature representation. Following Panoptic-PHNet~\cite{PHNet}, we leverage a Unet-based network with four encoding layers and four decoding layers as a semantic-segmentation task backbone with two decoder heads. Compared with other panoptic-segmentation networks using completely different decoding modules, the first three decoding layers in our network are shared. We concatenate the fine-grained fused features and 3D-branch output features as the final representation of the voxel to conduct KNN and hypergraph learning. Finally, the prediction of voxels maps back to the point level according to their position with instance-label supervising.

\section{Experiment}

In this section, we present our panoptic-segmentation results on the SemanticKITTI~\cite{kitti} dataset \textcolor{black}{and nuScenes~\cite{nuscenes} dataset} first. Then, we discuss the influence of the hypergraph-learning module and the selection of cluster algorithms in our proposed framework.

\subsection{Datasets and Loss Function}

\textbf{SemanticKITTI} is the first benchmark that presents challenges for the LiDAR panoptic-segmentation task~\cite{kitti}. It was created by annotating the KITTI odometry dataset~\cite{semantickitti}, which consists of 22 sequences captured using a 64-beam LiDAR sensor. Of these sequences, 10 were used for training (19,130 training frames and 4,071 validation frames); 11, for testing; and 1, for validation (20,351 frames in total). The dataset provides annotated point-wise labels for 20 classes for segmentation tasks, eight of which are defined as ``thing" classes.

\textcolor{black}{\textbf{nuScenes} is a large-scale multimodal dataset for autonomous driving. It contains a 32-beam LiDAR, five radars, six RGB cameras, and maps, covering 1000 real-world driving scenes from four locations in Boston and Singapore~\cite{nuscenes}. There are 850 annotated scenes for training and 150 for testing. The panoptic annotations contain 10 ``thing" classes, six ``stuff" classes, and one class for noisy labels.}

\textbf{Evaluation metric} As first proposed in Panoptic Segmentation~\cite{panoptic}, we used panoptic quality (PQ), segmentation quality (SQ), and recognition quality (RQ) to evaluate panoptic segmentation. To further discuss our method's performance, we calculated $P{Q^{St}}$, $S{Q^{St}}$, $R{Q^{St}}$ for ``stuff" classes and $P{Q^{Th}}$, $S{Q^{Th}}$, and $R{Q^{Th}}$ for ``thing" classes separately. According to SemanticKITTI panoptic segmentation requirement, PQ could be further deconstructed into PQ as follows:

\begin{equation}

P{Q_c} = \underbrace {\frac{{\sum\nolimits_{(p,q) \in T{P_c}} {IoU(p,q)} }}{{\left| {T{P_c}} \right|}}}_{SQ} \times \underbrace {\frac{{\left| {T{P_c}} \right|}}{{\left| {T{P_c}} \right| + \frac{1}{2}\left| {F{P_c}} \right| + \frac{1}{2}\left| {F{N_c}} \right|}}}_{RQ},

\end{equation}

\begin{equation}

PQ = \frac{1}{n}\sum\limits_{c = 1}^n {P{Q_c}}.

\end{equation}

The first part is SQ, and the second part is RQ, where n denotes the total number of labeled classes, and $(p,q)$ represents the prediction and ground truth. ${\left| {T{P_c}} \right|}$, ${\left| {F{P_c}} \right|}$, and ${\left| {F{N_c}} \right|}$ are set of true-positive, false-positive, and false-negative matches for class c.

\textbf{Training set and loss Function.} In our experiments, we used the SGD optimizer with a momentum of 0.9, a learning rate of 0.001, and a weight decay of 0.0005 to train our HyperG-PS model. \textcolor{black}{As the performance of semantic segmentation is crucial to the panoptic task, we trained the semantic head separately for 40 epochs for the SemanticKITTI dataset~\cite{kitti} and 25 epochs for the nuScenes dataset~\cite{nuscenes} before starting the full training process, respectively. Afterward, we trained instance heads and semantic heads together for another 50 epochs, and the two branches shared their parameters and leveraged hyper-parameters to concentrate on the instance head. During the training, we adopted cross-entropy loss for the semantic task ($L_{sem}$) and the hypergraph-learning process ($L_{ins}$). We also followed~\cite{blovasz} by utilizing Lovazs softmax loss ($L_{ls}$) in the semantic branch. The final loss function can be denoted as

\begin{equation}

L = \alpha{L_{sem}} + \lambda{L_{ins}} + {L_{ls}},

\end{equation}

where $\alpha=0.2$ and $\lambda=0.8$ are semantic minimizing factors that further improve the model performance in the instance-segmentation period.} In the experiment, we applied data augmentation as PolarNet~\cite{polarnet} for datasets with a limited number of moving instances during the instance training period. All experiments were conducted on NVIDIA RTX 3090 GPUs.

\subsection{Main Results}

\textbf{Quantitative analysis}. Table~\ref{tab1} shows the quantitative experimental results on the SemanticKITTI validation set. We compared our approach with state-of-the-art panoptic-segmentation approaches on the SemanticKITTI validation set~\cite{kitti}. Our proposed method was compared with the state-of-the-art methods and outperformed them in terms of the overall PQ and RQ. \textcolor{black}{Specifically, our method demonstrated an improvement of 1.1\% in overall PQ compared with GP-S3Net~\cite{GP}, and the simple-graph-based LiDAR panoptic-segmentation method, which remained at the top of the SemanticKITTI~\cite{kitti} leader board. Compared with other methods in Table~\ref{tab1}, our method showed its good performance in ``thing" classes PQ with 1.4\%, which is a more challenging part of the panoptic-segmentation task and more important for potential application in the autonomous driving field. The PQ of the ``thing" class largely depends on voxel-clustering results, which reveals the effectiveness of our proposed hypergraph-based method. Even though our proposed framework was not as good as Panoptic-PHnet in SQ, according to Equation (4), it is more convincing that our hypergraph structure has a great advantage in modeling the correlation between the specific object and its ID. Moreover, compared with transformer-based methods EfficientLPS~\cite{lps} and Panoptic-PHnet~\cite{PHNet}, our hypergraph-based approach outperformed approximately 17\% on ``thing" classes while falling short 2.1\% and 1.1\% on ``stuff" classes. These results also reveal that hypergraph learning has superior capability to construct the relationship between an ID label and object point-cloud features, whereas transformer-based methods enhance the point-cloud features directly.} We can further discuss the function of the hypergraph-learning module in the next section.

Table 2 demonstrates our proposed method's performance on the nuScenes validation set~\cite{nuscenes}. Given that the 32-beam LiDAR utilized in the nuScenes dataset makes instance point-cloud features less prominent, it poses a greater challenge for panoptic segmentation. The proposed method surpassed all state-of-the-art methods in overall PQ and RQ, showing advantages in the performance of “thing” classes. As regards the nuScene dataset~\cite{nuscenes}, our approach demonstrated a performance improvement of 0.7\% in PQ and 2.7\%in PQTh over the transformer-based method Panoptic-PHnet~\cite{PHNet} while showing a decrease of 1.6\% in PQSt. Compared with other graph-based methods such as GP-S3Net~\cite{GP}, our method achieved a significant increase of over 7.4\% in PQ and 8.5\% PQTh, respectively. The experimental results on SemanticKITTI~\cite{kitti} and nuScenes~\cite{nuscenes} show that the hypergraph-based method has great advantages in modeling the correlation between the object features and instance ID, which is suitable for the panoptic-segmentation task.

%==================tab==================%

\begin{table}[]

\begin{center}

\caption{Comparison of LiDAR panoptic-segmentation performance on SemanticKITTI validation dataset~\cite{kitti}. \textcolor{black}{The metric is in [\%]. We observed that our proposed methods were 1.1\% and 2.6\% better than the state-of-the-art methods GP-S3Net~\cite{GP} and Panoptic-PHnet~\cite{PHNet} in the overall PQ, respectively.}}

\vspace{0.2cm}

\label{tab1}

% \resizebox{\linewidth}{27mm}{

\small

\setlength{\tabcolsep}{0.9mm}{

\resizebox{\textwidth}{30mm}{

\begin{tabular}{llllllllll}

\toprule[2pt]

\textcolor{black}{Method }&\textcolor{black}{PQ }& \textcolor{black}{RQ }&\textcolor{black}{SQ }&\textcolor{black}{$PQ^{Th}$}&\textcolor{black}{$RQ^{Th}$}&\textcolor{black}{$SQ^{Th}$}&\textcolor{black}{$PQ^{St}$}&\textcolor{black}{$RQ^{St}$}&\textcolor{black}{$SQ^{St}$} \\\hline

\textcolor{black}{RangeNet++~\cite{rangenet++} }&\textcolor{black}{37.1}& \textcolor{black}{47.2}&\textcolor{black}{75.9}&\textcolor{black}{20.2}&\textcolor{black}{25.2}&\textcolor{black}{75.2}&\textcolor{black}{49.3}&\textcolor{black}{62.8 }&\textcolor{black}{76.5} \\

\textcolor{black}{LPSAD~\cite{milioto} }&\textcolor{black}{38.0}& \textcolor{black}{48.2}&\textcolor{black}{76.5}&\textcolor{black}{25.6}&\textcolor{black}{31.8}&\textcolor{black}{76.8}&\textcolor{black}{47.1}&\textcolor{black}{60.1 }&\textcolor{black}{76.2} \\

\textcolor{black}{KPConv+P.P.~\cite{pointpillars} }&\textcolor{black}{44.5}& \textcolor{black}{54.4}&\textcolor{black}{80.2}&\textcolor{black}{32.7}&\textcolor{black}{38.7}&\textcolor{black}{81.5}&\textcolor{black}{53.1}&\textcolor{black}{65.9 }&\textcolor{black}{79.0} \\

\textcolor{black}{Panoster~\cite{panoster} }&\textcolor{black}{52.7}& \textcolor{black}{64.1}&\textcolor{black}{80.7}&\textcolor{black}{49.5}&\textcolor{black}{58.5}&\textcolor{black}{83.3}&\textcolor{black}{55.3}&\textcolor{black}{68.3 }&\textcolor{black}{78.8} \\

\textcolor{black}{Panoptic-PolarNet~\cite{polarnet}}&\textcolor{black}{54.1}& \textcolor{black}{65.2}&\textcolor{black}{81.4}&\textcolor{black}{53.3}&\textcolor{black}{60.6}&\textcolor{black}{\textBF{87.3}}&\textcolor{black}{55.1 }&\textcolor{black}{68.2 }&\textcolor{black}{77.3 } \\

\textcolor{black}{DS-Net~\cite{DSN} }&\textcolor{black}{57.7}& \textcolor{black}{68.0}&\textcolor{black}{77.6}&\textcolor{black}{55.1}&\textcolor{black}{68.8}&\textcolor{black}{78.2 }&\textcolor{black}{54.8}&\textcolor{black}{67.3}&\textcolor{black}{77.1} \\

\textcolor{black}{EfficientLPS~\cite{lps} }&\textcolor{black}{59.2}& \textcolor{black}{69.8}&\textcolor{black}{75.3}&\textcolor{black}{58.1}&\textcolor{black}{68.2}&\textcolor{black}{78.8 }&\textcolor{black}{\textBF{60.9} }&\textcolor{black}{71.0}&\textcolor{black}{72.8} \\

\textcolor{black}{Panoptic-PHnet~\cite{PHNet} }&\textcolor{black}{61.7}& \textcolor{black}{72.1}&\textcolor{black}{\textBF{85.8}}&\textcolor{black}{69.3}&\textcolor{black}{70.4}&\textcolor{black}{86.7 }&\textcolor{black}{59.9 }&\textcolor{black}{\textBF{73.3}}&\textcolor{black}{80.5} \\

\textcolor{black}{GP-S3Net~\cite{GP} }&\textcolor{black}{63.2}& \textcolor{black}{75.9}&\textcolor{black}{81.4 }&\textcolor{black}{70.2}&\textcolor{black}{86.2}&\textcolor{black}{80.1}&\textcolor{black}{58.3}&\textcolor{black}{71.9}&\textcolor{black}{77.9} \\ \hline

\textcolor{black}{\textBF{HyperG-PS (ours)} }&\textcolor{black}{\textBF{64.3}}& \textcolor{black}{\textBF{76.5}}&\textcolor{black}{83.8}&\textcolor{black}{\textBF{71.7}}&\textcolor{black}{\textBF{87.4}}&\textcolor{black}{82.8}&\textcolor{black}{58.8 }&\textcolor{black}{73.2}&\textcolor{black}{\textBF{80.9}} \\

\bottomrule[2pt]

\end{tabular}

} }

\vspace{-0.5cm}

\end{center}

\end{table}

%=======================================%

%==================tab==================%

% \begin{table}[]

% \begin{center}

% \caption{Comparison of LiDAR panoptic-segmentation performance on SemanticKITTI validation dataset. \textcolor{black}{The metric is in [\%]. We can observe that our proposed methods are 1.1\% and 2.6\% better than the state-of-the-art methods GP-S3Net~\cite{GP} and Panoptic-PHnet~\cite{PHNet} on overall panoptic quality (PQ), respectively.}}

% \vspace{0.2cm}

% \label{tab1}

% % \resizebox{\linewidth}{27mm}{

% \small

% \setlength{\tabcolsep}{0.9mm}{

% \resizebox{\textwidth}{30mm}{

% \begin{tabular}{llllllllll}

% \toprule[2pt]

% \textcolor{blue}{Method }&\textcolor{blue}{PQ }& \textcolor{blue}{RQ }&\textcolor{blue}{SQ }&\textcolor{blue}{$PQ^{Th}$}&\textcolor{blue}{$RQ^{Th}$}&\textcolor{blue}{$SQ^{Th}$}&\textcolor{blue}{$PQ^{St}$}&\textcolor{blue}{$RQ^{St}$}&\textcolor{blue} {$SQ^{St}$}\\\hline

% \textcolor{blue}{RangeNet++~\cite{rangenet++} }&\textcolor{blue}{37.1}& \textcolor{blue}{47.2}&\textcolor{blue}{75.9}&\textcolor{blue}{20.2}&\textcolor{blue}{25.2}&\textcolor{blue}{75.2}&\textcolor{blue}{49.3}&\textcolor{blue}{62.8 }&\textcolor{blue}{76.5} \\

% \textcolor{blue}{LPSAD~\cite{milioto} }&\textcolor{blue}{38.0}& \textcolor{blue}{48.2}&\textcolor{blue}{76.5}&\textcolor{blue}{25.6}&\textcolor{blue}{31.8}&\textcolor{blue}{76.8}&\textcolor{blue}{47.1}&\textcolor{blue}{60.1 }&\textcolor{blue}{76.2} \\

% \textcolor{blue}{KPConv+P.P.~\cite{pointpillars} }&\textcolor{blue}{44.5}& \textcolor{blue}{54.4}&\textcolor{blue}{80.2}&\textcolor{blue}{32.7}&\textcolor{blue}{38.7}&\textcolor{blue}{81.5}&\textcolor{blue}{53.1}&\textcolor{blue}{65.9 }&\textcolor{blue}{79.0} \\

% \textcolor{blue}{Panoster~\cite{panoster} }&\textcolor{blue}{52.7}& \textcolor{blue}{64.1}&\textcolor{blue}{80.7}&\textcolor{blue}{49.5}&\textcolor{blue}{58.5}&\textcolor{blue}{83.3}&\textcolor{blue}{55.3}&\textcolor{blue}{68.3 }&\textcolor{blue}{78.8} \\

% \textcolor{blue}{Panoptic-PolarNet~\cite{polarnet}}&\textcolor{blue}{54.1}& \textcolor{blue}{65.2}&\textcolor{blue}{81.4}&\textcolor{blue}{53.3}&\textcolor{blue}{60.6}&\textcolor{blue}{\textBF{87.3}}&\textcolor{blue}{55.1 }&\textcolor{blue}{68.2 }&\textcolor{blue}{77.3 } \\

% \textcolor{blue}{DS-Net~\cite{DSN} }&\textcolor{blue}{57.7}& \textcolor{blue}{68.0}&\textcolor{blue}{77.6}&\textcolor{blue}{55.1}&\textcolor{blue}{68.8}&\textcolor{blue}{78.2 }&\textcolor{blue}{54.8}&\textcolor{blue}{67.3}&\textcolor{blue}{77.1} \\

% \textcolor{blue}{EfficientLPS~\cite{lps} }&\textcolor{blue}{59.2}& \textcolor{blue}{69.8}&\textcolor{blue}{75.3}&\textcolor{blue}{58.1}&\textcolor{blue}{68.2}&\textcolor{blue}{78.8 }&\textcolor{blue}{\textBF{60.9} }&\textcolor{blue}{71.0}&\textcolor{blue}{72.8} \\

% \textcolor{blue}{Panoptic-PHnet~\cite{PHNet} }&\textcolor{blue}{61.7}& \textcolor{blue}{72.1}&\textcolor{blue}{\textBF{85.8}}&\textcolor{blue}{69.3}&\textcolor{blue}{70.4}&\textcolor{blue}{86.7 }&\textcolor{blue}{59.9 }&\textcolor{blue}{\textBF{73.3}}&\textcolor{blue}{80.5} \\

% \textcolor{blue}{GP-S3Net~\cite{GP} }&\textcolor{blue}{63.2}& \textcolor{blue}{75.9}&\textcolor{blue}{81.4 }&\textcolor{blue}{70.2}&\textcolor{blue}{86.2}&\textcolor{blue}{80.1}&\textcolor{blue}{58.3}&\textcolor{blue}{71.9}&\textcolor{blue}{77.9} \\ \hline

% \textcolor{blue}{\textBF{HyperG-PS (ours)} }&\textcolor{blue}{\textBF{64.3}}& \textcolor{blue}{\textBF{76.5}}&\textcolor{blue}{83.8}&\textcolor{blue}{\textBF{71.7}}&\textcolor{blue}{\textBF{87.4}}&\textcolor{blue}{82.8}&\textcolor{blue}{58.8 }&\textcolor{blue}{73.2}&\textcolor{blue}{\textBF{80.9}} \\

% \bottomrule[2pt]

% \end{tabular}

% } }

% \vspace{-0.5cm}

% \end{center}

% \end{table}

%=======================================%

%==================tab==================%

\begin{table}[]

\begin{center}

\caption{\textcolor{black}{Comparison of LiDAR panoptic-segmentation performance on the nuScene validation dataset. The metric is in [\%]. We observed that our proposed methods were 0.7\% better than the state-of-the-art methods PUPS~\cite{pups} and Panoptic-PHnet~\cite{PHNet} in the overall PQ.}}

\vspace{0.2cm}

\label{tab2}

% \resizebox{\linewidth}{27mm}{

\small

\setlength{\tabcolsep}{0.9mm}{

\resizebox{\textwidth}{24.6mm}{

\begin{tabular}{llllllllll}

\toprule[2pt]

% \textcolor{blue}{Method} &\textcolor{blue}{PQ} &\textcolor{blue}{RQ} &\textcolor{blue}{SQ} &\textcolor{blue}{$PQ^{Th}$} &\textcolor{blue}{$RQ^{Th}$ } & \textcolor{blue}{$SQ^{Th}$} &\textcolor{blue}{$PQ^{St}$} &\textcolor{blue}{$RQ^{St}$} &\textcolor{blue}{$SQ^{St}$ } \\ \hline

\textcolor{black}{Method} &\textcolor{black}{PQ} &\textcolor{black}{RQ} &\textcolor{black}{SQ} &\textcolor{black}{$PQ^{Th}$} &\textcolor{black}{$RQ^{Th}$ } & \textcolor{black}{$SQ^{Th}$} &\textcolor{black}{$PQ^{St}$} &\textcolor{black}{$RQ^{St}$} &\textcolor{black}{$SQ^{St}$ } \\ \hline

\textcolor{black}{Panoptic TrackNet~\cite{rangenet++}}&\textcolor{black}{57.4}&\textcolor{black}{65.3}&\textcolor{black}{73.2}&\textcolor{black}{51.8}&\textcolor{black}{55.9}&\textcolor{black}{83.4}&\textcolor{black}{63.4}&\textcolor{black}{78.6}&\textcolor{black}{78.7}\\

\textcolor{black}{DS-Net~\cite{DSN} } &\textcolor{black}{59.9}&\textcolor{black}{69.7}&\textcolor{black}{78.3}&\textcolor{black}{56.1}&\textcolor{black}{62.8}&\textcolor{black}{73.4}&\textcolor{black}{62.1}&\textcolor{black}{74.5}&\textcolor{black}{79.3} \\

\textcolor{black}{GP-S3Net~\cite{GP} } &\textcolor{black}{66.9}&\textcolor{black}{75.9}&\textcolor{black}{80.4}&\textcolor{black}{68.2}&\textcolor{black}{71.2}&\textcolor{black}{87.1}&\textcolor{black}{66.3}&\textcolor{black}{80.9}&\textcolor{black}{83.1} \\

\textcolor{black}{EfficientLPS~\cite{lps} } &\textcolor{black}{67.1}&\textcolor{black}{76.7}&\textcolor{black}{85.1}&\textcolor{black}{63.1}&\textcolor{black}{62.5}&\textcolor{black}{85.8}&\textcolor{black}{71.5}&\textcolor{black}{84.6}&\textcolor{black}{83.7}\\

\textcolor{black}{Panoptic-PolarNet~\cite{polarnet}} &\textcolor{black}{69.4}&\textcolor{black}{81.3}&\textcolor{black}{85.4}&\textcolor{black}{66.3}&\textcolor{black}{74.6}&\textcolor{black}{87.5}&\textcolor{black}{72.1}&\textcolor{black}{85.2}&\textcolor{black}{83.6} \\

\textcolor{black}{PUPS~\cite{pups} } &\textcolor{black}{74.7}&\textcolor{black}{83.3}&\textcolor{black}{\textBF{89.4}} &\textcolor{black}{75.6}&\textcolor{black}{82.4} & \textcolor{black}{\textBF{91.8}}&\textcolor{black}{73.6} & \textcolor{black}{85.6} & \textcolor{black}{85.3} \\

\textcolor{black}{Panoptic-PHnet~\cite{PHNet} } &\textcolor{black}{74.7}&\textcolor{black}{\textBF{84.2}}&\textcolor{black}{88.2} &\textcolor{black}{74.0}&\textcolor{black}{82.5}&\textcolor{black}{89.0 }&\textcolor{black}{\textBF{75.9}}&\textcolor{black}{86.9}&\textcolor{black}{\textBF{86.8}} \\

% \textcolor{blue}{Panoptic-PHnet~\cite{PHNet} } &\textcolor{blue}{74.7}&\textcolor{blue}{\textBF{84.2}}&\textcolor{black}{88.2} &\textcolor{black}{74.0}&\textcolor{black}{82.5}&\textcolor{black}{89.0 }&\textcolor{black}{\textBF{75.9}}&\textcolor{black}{86.9}&\textcolor{black}{\textBF{86.8}} \\

\hline

% \textcolor{blue}{\textBF{HyperG-PS (ours)} } &\textcolor{black}{\textBF{75.4}}&\textcolor{blue}{\textBF{84.2}}&\textcolor{blue}{89.2}&\textcolor{blue}{\textBF{76.7}}&\textcolor{blue}{\textBF{84.2}} &\textcolor{blue}{90.4} &\textcolor{blue}{74.3} &\textcolor{blue}{\textBF{87.1}} &\textcolor{blue}{85.1} \\

\textcolor{black}{\textBF{HyperG-PS (ours)} } &\textcolor{black}{\textBF{75.4}}&\textcolor{black}{\textBF{84.2}}&\textcolor{black}{89.2}&\textcolor{black}{\textBF{76.7}}&\textcolor{black}{\textBF{84.2}} &\textcolor{black}{90.4} &\textcolor{black}{74.3} &\textcolor{black}{\textBF{87.1}} &\textcolor{black}{85.1} \\

\bottomrule[2pt]

\end{tabular}

}}

\vspace{-0.5cm}

\end{center}

\end{table}

%=======================================%

%==================tab==================%

% \begin{table}[]

% \begin{center}

% \caption{\textcolor{black}{Comparison of LiDAR panoptic-segmentation performance on nuScene validation dataset. The metric is in [\%]. We can observe that our proposed methods are 0.7\% better than the state-of-the-art methods PUPS~\cite{pups} and Panoptic-PHnet~\cite{PHNet} on overall panoptic quality (PQ).}}

% \vspace{0.2cm}

% \label{tab2}

% % \resizebox{\linewidth}{27mm}{

% \small

% \setlength{\tabcolsep}{0.9mm}{

% \resizebox{\textwidth}{24.6mm}{

% \begin{tabular}{llllllllll}

% \toprule[2pt]

% \textcolor{blue}{Method} &\textcolor{blue}{PQ} &\textcolor{blue}{RQ} &\textcolor{blue}{SQ} &\textcolor{blue}{$PQ^{Th}$} &\textcolor{blue}{$RQ^{Th}$ } & \textcolor{blue}{$SQ^{Th}$} &\textcolor{blue}{$PQ^{St}$} &\textcolor{blue}{$RQ^{St}$} &\textcolor{blue}{$SQ^{St}$ } \\ \hline

% % \textcolor{blue}{Method} &\textcolor{black}{PQ} &\textcolor{black}{RQ} &\textcolor{black}{SQ} &\textcolor{black}{$PQ^{Th}$} &\textcolor{black}{$RQ^{Th}$ } & \textcolor{black}{$SQ^{Th}$} &\textcolor{black}{$PQ^{St}$} &\textcolor{black}{$RQ^{St}$} &\textcolor{black}{$SQ^{St}$ } \\ \hline

% \textcolor{blue}{Panoptic TrackNet~\cite{rangenet++}}&\textcolor{blue}{57.4}&\textcolor{blue}{65.3}&\textcolor{blue}{73.2}&\textcolor{blue}{51.8}&\textcolor{blue}{55.9}&\textcolor{blue}{83.4}&\textcolor{blue}{63.4}&\textcolor{blue}{78.6}&\textcolor{blue}{78.7}\\

% \textcolor{blue}{DS-Net~\cite{DSN} } &\textcolor{blue}{59.9}&\textcolor{blue}{69.7}&\textcolor{blue}{78.3}&\textcolor{blue}{56.1}&\textcolor{blue}{62.8}&\textcolor{blue}{73.4}&\textcolor{blue}{62.1}&\textcolor{blue}{74.5}&\textcolor{blue}{79.3} \\

% \textcolor{blue}{GP-S3Net~\cite{GP} } &\textcolor{blue}{66.9}&\textcolor{blue}{75.9}&\textcolor{blue}{80.4}&\textcolor{blue}{68.2}&\textcolor{blue}{71.2}&\textcolor{blue}{87.1}&\textcolor{blue}{66.3}&\textcolor{blue}{80.9}&\textcolor{blue}{83.1} \\

% \textcolor{blue}{EfficientLPS~\cite{lps} } &\textcolor{blue}{67.1}&\textcolor{blue}{76.7}&\textcolor{blue}{85.1}&\textcolor{blue}{63.1}&\textcolor{blue}{62.5}&\textcolor{blue}{85.8}&\textcolor{blue}{71.5}&\textcolor{blue}{84.6}&\textcolor{blue}{83.7}\\

% \textcolor{blue}{Panoptic-PolarNet~\cite{polarnet}} &\textcolor{blue}{69.4}&\textcolor{blue}{81.3}&\textcolor{blue}{85.4}&\textcolor{blue}{66.3}&\textcolor{blue}{74.6}&\textcolor{blue}{87.5}&\textcolor{blue}{72.1}&\textcolor{blue}{85.2}&\textcolor{blue}{83.6} \\

% \textcolor{blue}{PUPS~\cite{pups} } &\textcolor{blue}{74.7}&\textcolor{blue}{83.3}&\textcolor{blue}{\textBF{89.4}} &\textcolor{blue}{75.6}&\textcolor{blue}{82.4} & \textcolor{blue}{\textBF{91.8}}&\textcolor{blue}{73.6} & \textcolor{blue}{85.6} & \textcolor{blue}{85.3} \\

% \textcolor{blue}{Panoptic-PHnet~\cite{PHNet} } &\textcolor{blue}{74.7}&\textcolor{blue}{\textBF{84.2}}&\textcolor{blue}{88.2} &\textcolor{blue}{74.0}&\textcolor{blue}{82.5}&\textcolor{blue}{89.0 }&\textcolor{blue}{\textBF{75.9}}&\textcolor{blue}{86.9}&\textcolor{blue}{\textBF{86.8}} \\

% % \textcolor{blue}{Panoptic-PHnet~\cite{PHNet} } &\textcolor{blue}{74.7}&\textcolor{blue}{\textBF{84.2}}&\textcolor{black}{88.2} &\textcolor{black}{74.0}&\textcolor{black}{82.5}&\textcolor{black}{89.0 }&\textcolor{black}{\textBF{75.9}}&\textcolor{black}{86.9}&\textcolor{black}{\textBF{86.8}} \\

% \hline

% \textcolor{blue}{\textBF{HyperG-PS (ours)} } &\textcolor{blue}{\textBF{75.4}}&\textcolor{blue}{\textBF{84.2}}&\textcolor{blue}{89.2}&\textcolor{blue}{\textBF{76.7}}&\textcolor{blue}{\textBF{84.2}} &\textcolor{blue}{90.4} &\textcolor{blue}{74.3} &\textcolor{blue}{\textBF{87.1}} &\textcolor{blue}{85.1} \\

% % \textcolor{black}{\textBF{HyperG-PS (ours)} } &\textcolor{black}{\textBF{75.4}}&\textcolor{black}{\textBF{84.2}}&\textcolor{black}{89.2}&\textcolor{black}{\textBF{76.7}}&\textcolor{black}{\textBF{84.2}} &\textcolor{black}{90.4} &\textcolor{black}{74.3} &\textcolor{black}{\textBF{87.1}} &\textcolor{black}{85.1} \\

% \bottomrule[2pt]

% \end{tabular}

% }}

% \vspace{-0.5cm}

% \end{center}

% \end{table}

%=======================================%

%==================fig==================%

\begin{figure*}[t]

\center

\includegraphics[width=1\textwidth]{picture/pic6.png}

\vspace{-0.5cm}

\caption{\textcolor{black}{Visualization comparison of LiDAR panoptic-segmentation results with EfficientLPS~\cite{lps} and Panopic-PHNet~\cite{PHNet} and GP-S3Net~\cite{GP} on the SemanticKITTI~\cite{kitti} validation set. The blank parts were enlarged four times, demonstrating our method's superior performance at long distances and in overlap situations.}}

\vspace{-0.3cm}

\label{fig3}

\end{figure*}

%=======================================%

\textcolor{black}{\textbf{Qualitative Analysis}. Figure~\ref{fig3} and Figure~\ref{fig4} demonstrate the visualization of panoptic-segmentation results on the SemanticKITTI~\cite{kitti} and nuScenes~\cite{nuscenes} datasets, respectively. The results generated by Panopic-PolarNet~\cite{polarnet}, EfficientLPS~\cite{lps}, GP-S3Net~\cite{GP}, and our proposed method are visualized from left to right. As a visual comparison on SemanticKITTI~\cite{kitti}, shown in Figure~\ref{fig3}, our proposed method could generate relatively satisfactory segmentation, even at the long distance and with large overlap between two instances. These methods we compared our method with are all based on point offset predictions and clustering, and they performed poorly in crowded scenes, failing to differentiate between instances of pedestrians and vehicles, especially when they were close to the LiDAR view. In contrast, our proposed method enhanced voxel feature representation to separate most of the objects, regardless of their closeness, with less difficulty in clustering operation. As shown in the qualitative comparison on nuScenes~\cite{nuscenes} in Figure~\ref{fig4}, our method had superior performance even with sparse LiDAR point clouds at close instances, thanks to a hypergraph structure that can model the correlation between a specific instance and their ID. We utilize the cross-entropy loss in the hypergraph-learning module for instance segmentation, which constrains the point cloud effectively. }

%==================fig==================%

\begin{figure*}[!h]

\center

\includegraphics[width=1\textwidth]{picture/pic5.png}

\vspace{-0.5cm}

\caption{\textcolor{black}{Visualization comparison of LiDAR panoptic-segmentation results with EfficientLPS~\cite{lps} and Panopic-PHNet~\cite{PHNet} and GP-S3Net~\cite{GP} on the nuScenes~\cite{nuscenes} validation set. The blank parts were enlarged four times, demonstrating our method's good performance on the sparse point cloud.}}

\vspace{-0.3cm}

\label{fig4}

\end{figure*}

%=======================================%

%==================fig==================%

\begin{figure*}[t]

\center

\includegraphics[width=1\textwidth]{picture/pic33.png}

\vspace{-0.3cm}

\caption{Ablation study on the SemanticKITTI~\cite{kitti} validation set. (a) The network benefits from the hypergraph-learning module. (b) Addition of our proposed hypergraph-learning module on other cluster-based instance-segmentation methods, with the network showing a BEV better than that using RV.}

\vspace{-0.3cm}

\label{fig5}

\end{figure*}

%=======================================%

\subsection{Ablation Study}

\textbf{Ablation on hypergraph-learning module.}

We began by analyzing the effects of the proposed hypergraph-learning module in our framework for the task. We adopted the framework of our method without the hypergraph-learning module as the baseline.

As Figure~\ref{fig5}(a) shows, the hypergraph-learning module had significant effects on PQ and $PQ^{Th}$. Since our backbone fuses both 2D and 3D features, without the hypergraph-learning module, it still had a relatively strong capability for voxel representation for segmentation tasks in ``stuff" classes. Nevertheless, the network still requires the hypergraph-learning module to provide further features enhanced for voxels, particularly on instance segmentation in ``thing" classes. In other words, the ablation experiment demonstrated that the hypergraph-learning module mainly improved voxel features in the instance with a specific label that provides a hypergraph structure ground truth. PQ in the ``thing" class increased almost 10\% (9.9\%) in ``thing" classes, while it only showed a slight improvement (3.5\%) in the stuff classes.

Moreover, we applied our hypergraph-learning module to other cluster-based methods. The result in Figure~\ref{fig4} (b) indicates that DS-Net~\cite{DSN} benefited greatly, showing an overall PQ of 4.2\%, while EfficientLPS~\cite{lps} increased 1.2\%. That might be because the EfficientLPS adopts the RV, rather than BEV, to extract low-level features, which have a completely different feature pattern from our network. Compared with EfficientLPS, DS-Net is similar to our proposed method, which also adopts BEV and polar coordinates to balance the distribution of the LiDAR point cloud. The result of $PQ^{Th}$ in the ``thing" classes also shows that the hypergraph-learning modules still work if an instance-label-based hypergraph structure exits.

\textbf{Ablation on the selection of the cluster algorithms.}

%==================tab==================%

\begin{table}[]

\begin{center}

\caption{Ablation study on the selection of the cluster algorithms evaluated on the SemanticKITTI ~\cite{kitti}validation datasets. The result indicates that KNN-based cluster algorithms are most suitable for our framework.}

\label{tab3}

\vspace{0.2cm}

% \resizebox{110mm}{14mm}{

\begin{tabular}{lllllllllll}

\toprule[2pt]

Framework & PQ & $PQ^{Th}$ & $RQ^{Th}$ & $SQ^{Th}$ \\\hline

Baseline w/DBSCAN~\cite{DBSCAN} & 52.1& 47.2& 58.9 & 78.2 \\

Baseline w/HDBSCAN~\cite{HDBS} & 54.0& 52.2& 68.5 & 78.6 \\

Baseline w/MeanShift\cite{MS} & 59.3 & 64.7& 79.2 & 81.7 \\\hline

\textbf{Ours w/KNN} & \textBF{64.3}& \textBF{71.7}& \textBF{87.4} & \textBF{82.8} \\

\bottomrule[2pt]

\end{tabular}

% }

\vspace{-0.5cm}

\end{center}

\end{table}

%=======================================%

In our proposed method, the effectiveness of the network is largely determined by the cluster algorithms. Thus, we utilize three widely used cluster algorithms---DBSCAN~\cite{DBSCAN}, HDBSCAN~\cite{HDBS}, and MeanShift~\cite{MS}---to replace KNN algorithms in our proposed hypergraph-learning module. The baseline is our fusion backbone for semantic segmentationm followed by a hypergraph-learning module with different algorithms. Table~\ref{tab3} shows that distance-based algorithms have much better performance than density-based ones because the LiDAR point cloud has a highly irregular and sparse distribution. The other reason is that the hypergraph-learning module utilizes a change of distance to enhance the representation of the voxel, while in density-based methods, the change of density is difficult to spread to the voxel feature. As a distance-based method, MeanShift showed significantly better performance. In general, the proposed method outperformed all the baselines by a large margin.

\textcolor{black}{\textbf{Ablation with the transformer-based method.}

As stated in section 4.2, transformer-based approaches demonstrate an advantage in directly extracting point-cloud features for semantic-segmentation tasks. Similarly, our proposed hypergraph-based method excels in establishing correlations between instance IDs and original features, resulting in improved performance in panoptic-segmentation tasks. If computational efficiency is not a concern, combining transformer and hypergraph techniques can achieve even better results in this task. We conducted an ablation experiment by combining the hypergraph-learning instance-segmentation heads with the transformer-based semantic-segmentation heads from the state-of-the-art transformer-based method Panoptic-PHNet~\cite{PHNet} on the SemanticKITTI validation dataset~\cite{kitti}. According to the result in Table~\ref{tab4}, this assembled model had superior performance in both semantic segmentation and panoptic segmentation. We also compared the result with another state-of-the-art assembled model, PUPS, which reflects a slight improvement.}

%==================tab==================%

\begin{table}[]

\begin{center}

\caption{\textcolor{black}{Ablation study on combination with transformer-based methods evaluated on SemanticKITTI ~\cite{kitti}validation datasets. The result shows that the assembled model had superior performance on all evaluation metrics.}}

\label{tab4}

\small

\setlength{\tabcolsep}{1mm}{

\resizebox{\textwidth}{15mm}{

\vspace{0.2cm}

% \resizebox{110mm}{14mm}{

\begin{tabular}{lllllllllll}

\toprule[2pt]

\textcolor{black}{Framework } & \textcolor{black}{ PQ } &\textcolor{black}{RQ } &\textcolor{black}{SQ } & \textcolor{black}{$PQ^{Th}$ }& \textcolor{black}{$SQ^{Th}$ }& \textcolor{black}{$PQ^{St}$ }& \textcolor{black}{$SQ^{St}$ }\\\hline

\textcolor{black}{P.PHNet~\cite{PHNet} } & \textcolor{black}{ 61.7 } &\textcolor{black}{72.1 } &\textcolor{black}{85.8 } & \textcolor{black}{69.3 }& \textcolor{black}{86.7 }& \textcolor{black}{59.9 }& \textcolor{black}{80.5 } \\

\textcolor{black}{HyperG-PS } & \textcolor{black}{ 64.3 } &\textcolor{black}{76.5 } &\textcolor{black}{83.8 } & \textcolor{black}{71.7 }& \textcolor{black}{82.8 }& \textcolor{black}{58.8 }& \textcolor{black}{80.9}\\

\textcolor{black}{PUPS(Assembled) } & \textcolor{black}{ 66.3 } &\textcolor{black}{75.9 } &\textcolor{black}{82.5 } & \textcolor{black}{\textBF{74.6} }& \textcolor{black}{\textBF{93.4} }& \textcolor{black}{60.1 }& \textcolor{black}{74.5}\\\hline

\textcolor{black}{\textBF{P.PHNet w/hypergraph} } & \textcolor{black}{ \textBF{66.4}} &\textcolor{black}{\textBF{77.3}} &\textcolor{black}{\textBF{85.9}} & \textcolor{black}{73.8 }& \textcolor{black}{86.8 }& \textcolor{black}{\textBF{60.3}}& \textcolor{black}{\textBF{81.2} } \\

\bottomrule[2pt]

% \toprule[2pt]

% \textcolor{blue}{Method } & \textcolor{blue}{ PQ } &\textcolor{blue}{RQ } &\textcolor{blue}{SQ } & \textcolor{blue}{$PQ^{Th}$ }& \textcolor{blue}{$SQ^{Th}$ }& \textcolor{blue}{$PQ^{St}$ }& \textcolor{blue}{$SQ^{St}$ }\\\hline

% \textcolor{blue}{P.PHNet~\cite{PHNet} } & \textcolor{blue}{ 61.7 } &\textcolor{blue}{72.1 } &\textcolor{blue}{85.8 } & \textcolor{blue}{69.3 }& \textcolor{blue}{86.7 }& \textcolor{blue}{59.9 }& \textcolor{blue}{80.5 } \\

% \textcolor{blue}{HyperG-PS } & \textcolor{blue}{ 64.3 } &\textcolor{blue}{76.5 } &\textcolor{blue}{83.8 } & \textcolor{blue}{71.7 }& \textcolor{blue}{82.8 }& \textcolor{blue}{58.8 }& \textcolor{blue}{80.9}\\

% \textcolor{blue}{PUPS(Assembled) } & \textcolor{blue}{ 66.3 } &\textcolor{blue}{75.9 } &\textcolor{blue}{82.5 } & \textcolor{blue}{\textBF{74.6} }& \textcolor{blue}{\textBF{93.4} }& \textcolor{blue}{60.1 }& \textcolor{blue}{74.5}\\\hline

% \textcolor{blue}{\textBF{P.PHNet w/hypergraph} } & \textcolor{blue}{ \textBF{66.4}} &\textcolor{blue}{\textBF{77.3}} &\textcolor{blue}{\textBF{85.9}} & \textcolor{blue}{73.8 }& \textcolor{blue}{86.8 }& \textcolor{blue}{\textBF{60.3}}& \textcolor{blue}{\textBF{81.2} } \\

% \bottomrule[2pt]

\end{tabular}

} }

\vspace{-0.5cm}

\end{center}

\end{table}

%=======================================%

%==================tab==================%

\begin{table}[]

\begin{center}

\caption{\textcolor{black}{Ablation study on other graph-based modules on SemanticKITTI~\cite{kitti}validation datasets. The result shows that the hypergraph-based module outperformed the graph-based method in all metrics.}}

\label{tab5}

\vspace{0.2cm}

\setlength{\tabcolsep}{1mm}{

\resizebox{\textwidth}{14mm}{

% \resizebox{110mm}{12mm}{

\begin{tabular}{lllllllllll}

\toprule[2pt]

\textcolor{black}{method } & \textcolor{black}{PQ }&\textcolor{black}{RQ }&\textcolor{black}{SQ }& \textcolor{black}{$PQ^{Th}$} &\textcolor{black}{ $SQ^{Th}$}&\textcolor{black}{$PQ^{St}$}& \textcolor{black}{$SQ^{St}$ } \\\hline

\textcolor{black}{Baseline } & \textcolor{black}{58.2}&\textcolor{black}{69.4}&\textcolor{black}{75.7}& \textcolor{black}{57.6 } &\textcolor{black}{ 78.4 }&\textcolor{black}{56.9 } &\textcolor{black}{ 77.8 } \\

\textcolor{black}{Baseline w/graph~\cite{GP} } & \textcolor{black}{62.1}&\textcolor{black}{74.6}&\textcolor{black}{81.2}& \textcolor{black}{69.8 } &\textcolor{black}{80.9 }&\textcolor{black}{57.6 } &\textcolor{black}{ 78.2 } \\\hline

\textcolor{black}{\textBF{Baseline w/hypergraph}} & \textcolor{black}{\textBF{64.3}}&\textcolor{black}{\textBF{76.5}}&\textcolor{black}{\textBF{83.8}}& \textcolor{black}{\textBF{71.7 } } &\textcolor{black}{\textBF{82.8 } }&\textcolor{black}{\textBF{58.8 } } &\textcolor{black}{ \textBF{80.9 } } \\

\bottomrule[2pt]

\end{tabular}

} }

\vspace{-0.5cm}

\end{center}

\end{table}

%=======================================%

\textcolor{black}{\textbf{Ablation with the graph-based method.}

We conducted an ablation study by selecting different graph structures to model the correlations between the voxels and instances. We adopted the framework of our method without the hypergraph-learning module as the baseline. We replaced our hypergraph module with a simple graph-learning module from the GP-S3Net~\cite{GP}. The result in Table~\ref{tab5} demonstrates that the graph-based module could also improve the baseline performance by approximately 4\%(3.9\%) in overall PQ. However, the simple graph module could only enhance the feature differences between voxels belonging to different instances, but they could not strengthen the feature coherence among voxels that share the same semantic information. The SQ in Table~\ref{tab5} makes it clear that the hypergraph-learning module was superior in modeling complicated correlations between the voxels and instances. }

%==================tab==================%

% \begin{table}[]

% \begin{center}

% \caption{Ablation study on other graph-based modules on SemanticKITTI~\cite{kitti}validation datasets. The result demonstrates that the hypergraph-based module outperforms the graph-based method in all metrics.}

% \label{tab5}

% \vspace{0.2cm}

% \setlength{\tabcolsep}{1.4mm}{

% % \resizebox{110mm}{12mm}{

% \begin{tabular}{lllllllllll}

% \toprule[2pt]

% \textcolor{blue}{method } & \textcolor{blue}{PQ }&\textcolor{blue}{RQ }&\textcolor{blue}{SQ }& \textcolor{blue}{$PQ^{Th}$} &\textcolor{blue}{ $SQ^{Th}$}&\textcolor{blue}{$PQ^{St}$}& \textcolor{blue}{$SQ^{St}$ } \\\hline

% \textcolor{blue}{Baseline } & \textcolor{blue}{58.2}&\textcolor{blue}{69.4}&\textcolor{blue}{75.7}& \textcolor{blue}{57.6 } &\textcolor{blue}{ 78.4 }&\textcolor{blue}{56.9 } &\textcolor{blue}{ 77.8 } \\

% \textcolor{blue}{Baseline w/graph~\cite{GP} } & \textcolor{blue}{62.1}&\textcolor{blue}{74.6}&\textcolor{blue}{81.2}& \textcolor{blue}{69.8 } &\textcolor{blue}{80.9 }&\textcolor{blue}{57.6 } &\textcolor{blue}{ 78.2 } \\\hline

% \textcolor{blue}{\textBF{Baseline w/hypergraph}} & \textcolor{blue}{\textBF{64.3}}&\textcolor{blue}{\textBF{76.5}}&\textcolor{blue}{\textBF{83.8}}& \textcolor{blue}{\textBF{71.7 } } &\textcolor{blue}{\textBF{82.8 } }&\textcolor{blue}{\textBF{58.8 } } &\textcolor{blue}{ \textBF{80.9 } } \\

% \bottomrule[2pt]

% \end{tabular}

% }

% \vspace{-0.5cm}

% \end{center}

% \end{table}

%=======================================%

\section{Conclusion}

This paper proposed a hypergraph-based bottom-up panoptic-segmentation framework, named HyperG-PS, focusing on improving cluster performance on a voxelized point cloud in instance segmentation, which constitutes the core problem of the LiDAR panoptic-segmentation task. Our proposed framework uses a multi-view feature-extraction network that fuses 3D point-cloud features and 2D BEV features at the voxel level. We use a hypergraph to model the correlation between voxels in an instance, bridging the gap between voxel features and instance labels. Through hypergraph learning, we enhance the representation of voxels, which directly improves clustering performance without predicting point cloud offsets. Experimental results demonstrate that our proposed HyperG-PS achieved strong performance on the widely used SemanticKITTI datasets~\cite{kitti}. Our ablation experimental results demonstrate the strong capability of our hypergraph-learning module in feature enhancement.

%\section*{Acknowledgments}

%This work was supported by the National Natural Science Funds %of China (Nos. ).

% \label{}

%% The Appendices part is started with the command \appendix;

%% appendix sections are then done as normal sections

%% \appendix

%% \section{}

%% \label{}

%% If you have bibdatabase file and want bibtex to generate the

%% bibitems, please use

%%

\bibliographystyle{elsarticle-num}

\bibliography{ref}

%% else use the following coding to input the bibitems directly in the

%% TeX file.

% \begin{thebibliography}{00}

%% \bibitem{label}

%% Text of bibliographic item

% \bibitem{}

% \end{thebibliography}

\end{document}

\endinput

%%

%% End of file `elsarticle-template-num.tex'.
