# Supplementary material for: HyperG-PS: Voxel correlation modeling via hypergraph for LiDAR panoptic segmentation
Source: Fundam Res. 2025 Jan 26;6(3):1727–35. doi: 10.1016/j.fmre.2024.03.033 (PMC13247466; doi:10.1016/j.fmre.2024.03.033)
Supplement: Supplementary Data S2 — Supplementary Raw Research Data. This is open data under the CC BY license http://creativecommons.org/licenses/by/4.0/ [file mmc2.pdf]

CERTIFICATE

OF ENGLISH LANGUAGE EDITING

HyperG-PS: Voxel Correlation Modeling via Hypergraph for LiDAR Panoptic Segmentation

Light-detection-and-ranging (LiDAR) point-cloud panoptic segmentation is a fundamental task in autonomous driving because it integrates the tasks of static environmental understanding and dynamic object identification, which have recently gained significant research interest. In this paper, we propose a bottom-up panoptic-segmentation framework based on hypergraph learning, named HyperG-PS, which addresses the core problem of LiDAR panoptic segmentation by improving the cluster performance of instance segmentation,. Our framework inputs the raw LiDAR point cloud and uses a multi-view feature-extraction network to fuse the 3D point-cloud features and 2D BEV features at the voxel level. Afterward, we model the correlation among voxels using a hypergraph to bridge the gap between voxel features and instance labels. We enhance the representation of a voxel, thus ...

This document certifies that the manuscript listed above was copy edited for English language by LetPub, with regard to grammar, punctuation, spelling, and clarity. Documents receiving this certification should be regarded as having undergone professional editorial revision for English language before submission. However, the authors may accept or reject LetPub's suggestions and changes at their own discretion and LetPub does not have editorial control over the submitted documents. Submitted documents may have new text that was not provided to LetPub for review. Please use the verification link below to determine the validity of the submitted version.

February 23, 2024

Date of Revision

LetPub

LetPub is an author service brand owned and operated by Accdon LLC.
Tel: 1-781-202-9968 Email: info@accdon.com
Address: 400 Fifth Ave, Suite 530, Waltham, MA 02451, United States

This manuscript has been individually edited for grammar, punctuation, spelling, and clarity. You may verify the authenticity of this certificate on our website (https://www.letpub.com/editorial-certificate) at any time using this manuscript's unique code: PRL_240219U546.

© 2024 Accdon, LLC.
